# P-1401. Post-Tuberculosis Sequelae and Emerging Challenges in Nepal: Addressing AI powered X-ray, Drug Resistance, Co-Infections, and Rehabilitation Needs

**DOI:** 10.1093/ofid/ofaf695.1588

**Published:** 2026-01-11

**Authors:** Shambhu Joshi, Prarambhika Khadka

**Affiliations:** Mahabaudha Medical Center, Dhangadhi, Seti, Nepal; Mahabaudha Medical Center, Dhangadhi, Seti, Nepal

## Abstract

**Background:**

Nepal which have open borders with India and China faces a high burden of tuberculosis (TB), with an annual incidence of 70,000 cases and 18,000 deaths. Despite advancements in treatment, the increasing misuse of over-the-counter antibiotics, including second-line TB drugs, has escalated drug resistance, complicating post-TB management. Co-infections such as HIV and hepatitis B, Leprosy, Post COVID-19 combined with risk factors like smoking, exacerbate post-TB outcomes.

**Methods:**

A retrospective analysis of data from the National Tuberculosis Program (NTP) (2079/80) and tertiary hospital registries was conducted. Epidemiological trends, nutritional status, and co-infection patterns (HIV, hepatitis B) etc were examined. Pulmonary rehabilitation programs, AI built X-Ray and psychosocial counseling interventions were evaluated for their outcomes. Behavioral data, including smoking prevalence, were reviewed from patient surveys.

**Results:**

The study found that 2,900 MDR-TB cases are reported annually, with an 82% treatment success rate. Co-infections with HIV (0.6%) and hepatitis B (4.5%) significantly increased disease severity. Smoking prevalence among TB patients was 27%, contributing to poor recovery outcomes. Nutritional deficiencies were present in 68% of TB patients, with BMI improvements of 2.1 points seen after targeted nutritional interventions, Case reporting 82% support by AI assisted X-rays. Pulmonary rehabilitation improved lung function by 30% and reduced dyspnea by 20%. Psychosocial counseling reduced anxiety and depression scores by 40%, aiding patient reintegration into society.

**Conclusion:**

The findings highlight the importance of integrating pulmonary rehabilitation, Telemedicine, wider use of AI assisted X-rays where Health assessment technology support, Vaccination, free National Health Insurance program, nutritional supplementation, and psychosocial support into post-TB care programs in Nepal. Addressing antibiotic misuse, co-infections, and smoking through policy interventions and patient-centered care models is critical for improving outcomes. These efforts can significantly enhance post-TB recovery and reduce the disease burden in resource-limited settings.

**Disclosures:**

All Authors: No reported disclosures

